# Supplementary material for: The longest-lasting 2023 western North American heat wave was fueled by the record-warm Atlantic Ocean
Source: Nat Commun. 2025 Jul 16;16:6544. doi: 10.1038/s41467-025-61859-y (PMC12267422; doi:10.1038/s41467-025-61859-y)
Supplement: Supplementary file 1 — Supplementary Information [file 41467_2025_61859_MOESM1_ESM.pdf]

Supplementary Materials

The longest-lasting 2023 western North American heat wave was fueled by the record-warm Atlantic Ocean.

Hosmay Lopez[1], Sang-Ki Lee[1], Robert West[2], Dongmin Kim[2], Liwei Jia[3]

[1]Atlantic Oceanographic and Meteorological Laboratory, NOAA, Miami, Florida, USA

[2]Cooperative Institute for Marine and Atmospheric Studies, University of Miami, Miami, Florida, USA

[3]Geophysical Fluid Dynamics Laboratory, National Oceanic and Atmospheric Administration, Princeton, NJ, United States

Included Materials:

- Definition of interbasin (Atlantic-Pacific) synergy
- Supplementary Figures 1 through 11
- Supplementary Table 1

Atlantic-Pacific synergy (non-linear interactions)

Using the AGCM sea surface temperature anomaly (SSTA) sensitivity experiments, any variable (e.g., heat wave days, number, surface temperature) response to the prescribed forcing can be extracted as follows:

The climatological occurrence of any event (heat wave number, days) is first extracted from the control (CTL) simulation (equation S1). Note that we obtained this climatology from the 300 year-long CTL simulation so as to get an accurate expected value in the absence of any SSTA forcing. Then, the occurrences of such events in the SSTA sensitivity experiments are thus governed by eq. S2 for the Atlantic, eq. S3 for the Pacific, and S4 for the GBL23 experiment. For the case of both Atlantic and Pacific SSTA forcing (i.e., the GBL23 experiment), a linear combination of ATL23 and PAC23 is not sufficient, given that there could be interbasin interactions between the ATL and PAC SST forcing, i.e., the Synergy term in eq. S4.

$$CTL = Climatological\ expected\ occurrences \qquad (eq. S1)$$

$$ATL23 = CTL\ + Atlantic\ SSTA\ sensitivity \qquad (eq. S2)$$

$$PAC23 = CTL\ + Pacific\ SSTA\ sensitivity \qquad (eq. S3)$$

$$GBL23 = CTL\ + Atlantic\ SSTA\ + Pacific\ SSTA\ sensitivities\ + Synergy \qquad (eq. S4)$$

Subtracting eq. S2 and S3 from S4, we obtain the interbasin synergy component:

$$Interbasin\ Synergy = GBL23 - ATL23 - PAC23 + CTL \qquad (eq. S5)$$

Thus, the sum from ATL and PAC sensitivity experiments would result in the GBL23 minus the residual (i.e., synergic interbasin interactions) plus a climatology, which is present in all experiments.

Supplementary Figures

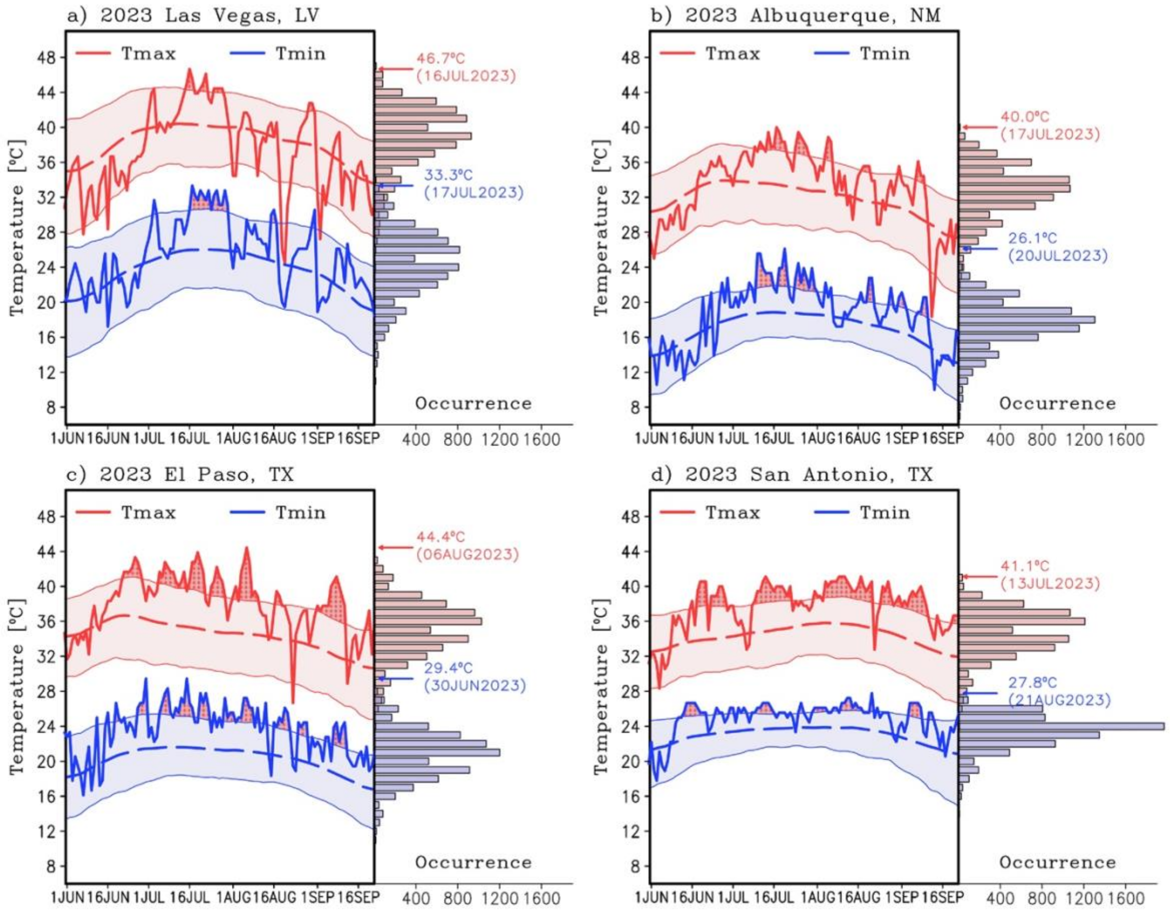

Supplementary Figure 1. Heat wave event of 2023. Seasonal evolution of maximum (red) and minimum (blue) temperature for the year 2023 from 1 June to 20 September for a) Las Vegas, Nevada, b) Albuquerque, New Mexico, c) El Paso, Texas, and d) San Antonio, Texas. The long-term daily mean is shown by the dashed line whereas the 5th and 95th percentiles are shown by the shading region. Excess above the 95th percentile is shown by red shading for both maximum and minimum temperatures. The observed histogram of maximum (red) and minimum (blue) temperature for the period 1 June to 31 August from 1955-2023 is also shown on the right with bin size of 1°C. The extremely warm temperatures for the 2023 summer are shown for reference. Source data are provided as a Source Data file.

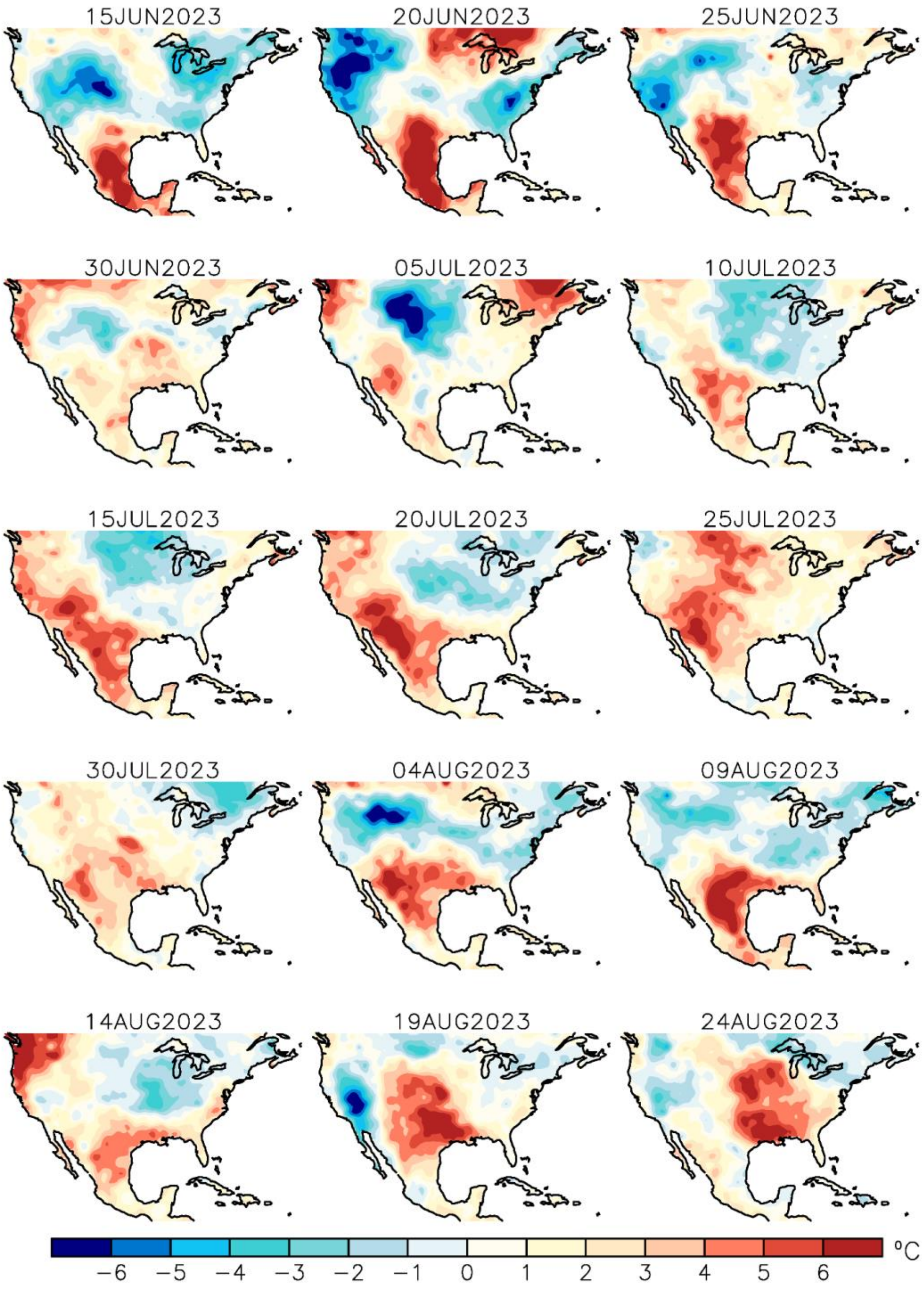

Supplementary Figure 2. Surface temperature anomaly evolution during the 2023 heatwave event averaged every pentad (five days centered at the labeled day) from 15 June to 24 August 2023.

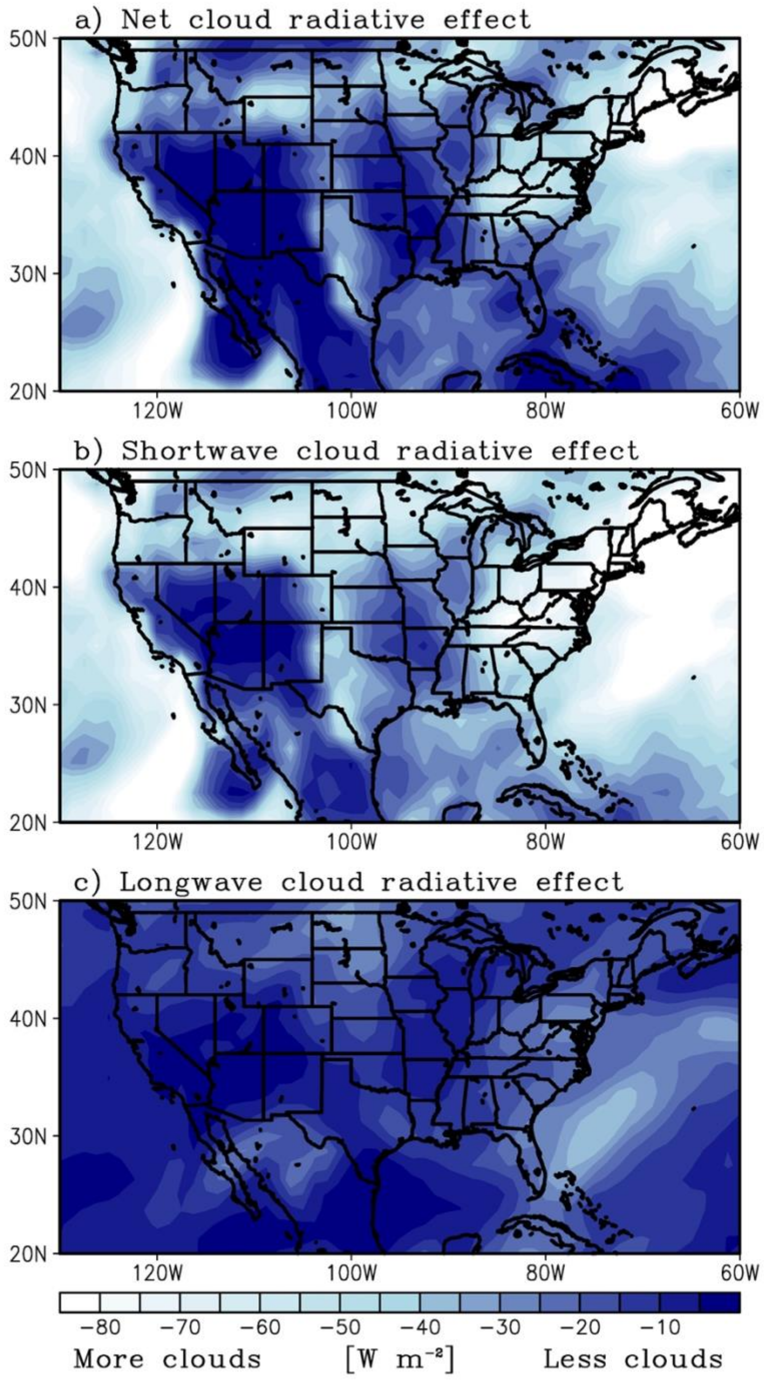

Supplementary Figure 3. Cloud radiative effects computed from the differences between clear-sky minus all-sky radiative fluxes at the top of the atmosphere (TOA) for a) net fluxes, b) shortwave reflected fluxes, and c) outgoing longwave fluxes. These values represent the five-day average centered on 19 July 2023, during the middle of the heat wave. Regions with dark blue represent little or no clouds.

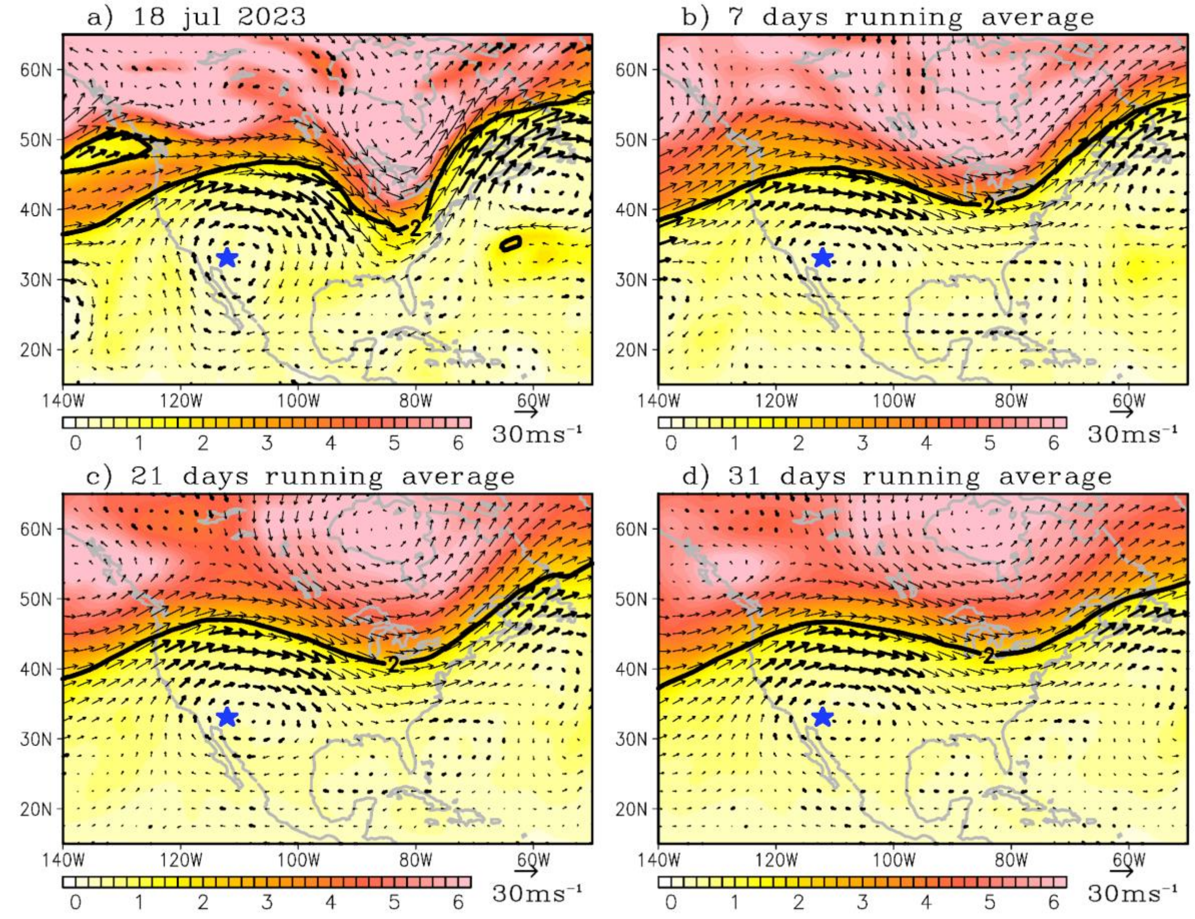

Supplementary Figure 4. a) Potential vorticity and wind at the 350K isentropic level during the maximum amplitude of the heat wave on 18 July 2023. Thick vectors depict anti-cyclonic fluid trapping, a proxy for heat dome and air flow stagnation. b), c), and d) are the same as a) but for the 7-, 21-, and 31-day running average centered on 18 July 2023. The thick black line indicates the location of the dynamical tropopause. The blue star on each panel represents the location of Phoenix, Arizona.

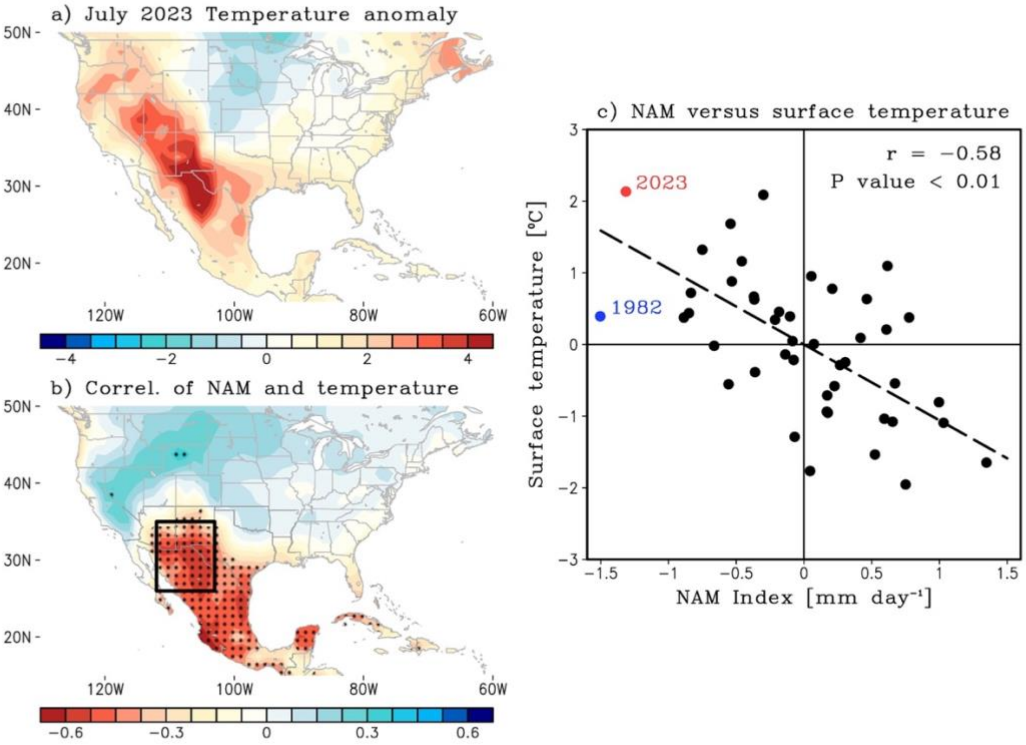

Supplementary Figure 5. a) July 2023 maximum 2-meter temperature anomalies. b) Correlation of maximum 2-meter temperatures and the North American Monsoon (NAM) index for the 1979 - 2022 period (the year 2023 was excluded from the correlation analysis intentionally to avoid aliasing the results). Note that the color bar is reversed for easier comparison and reflection of the negative correlations. c) Scatter plot of the NAM index versus 2-meter air temperatures averaged over the southwestern U.S. and northern Mexico (black box in panel b). The line of best fit is shown by the dashed line along with the years 1982 (blue) and 2023 (red). The correlation between the NAM and index and 2-m temperatures is r = -0.58, p-value < 0.01. See Methods for definition on the NAM index.

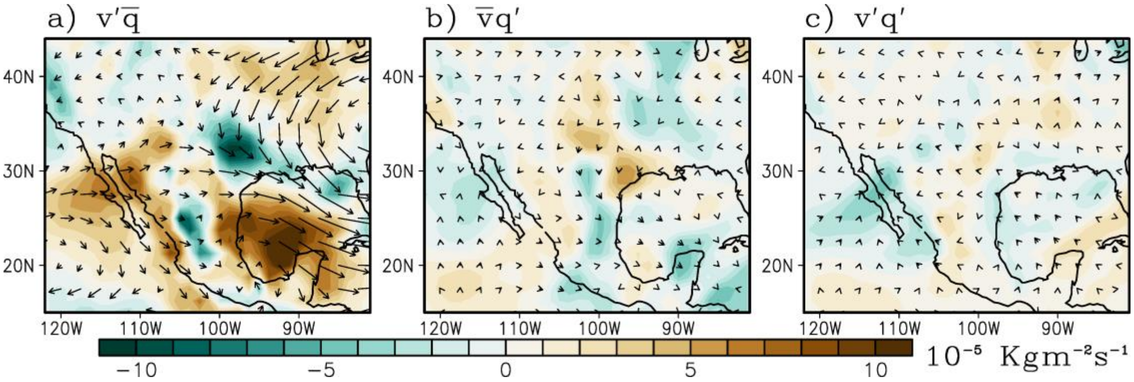

Supplementary Figure 6. Decomposition of the anomalous June-August 2023 vertically integrated moisture transport (vector, *Kg* m^{-1} s^{-1}) and its divergence (color, *Kg* m^{-2} s^{-1}), where negative values indicate convergence. a) Advection of mean moisture by anomalous wind, b) advection of anomalous moisture by the mean wind, and c) advection of anomalous moisture by

anomalous wind. The overbars denote climatology and primes denote deviation from climatology computed from the departure from the 1979-2022 climatology.

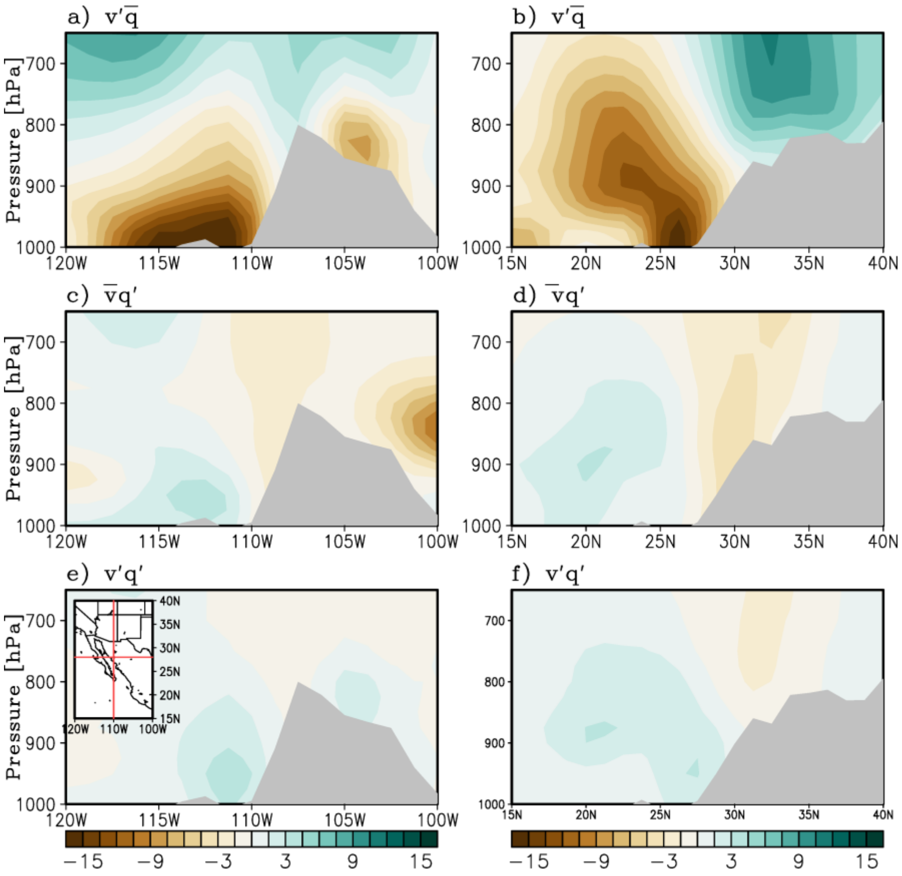

Supplementary Figure 7. (left-column) Zonal-vertical cross-section along 28°N of the 2023 moisture transport anomaly (color). (Right column) is the same but for the meridional cross-section along 110°W. The transport is decomposed into its components such as (a and b) advection of mean moisture by anomalous wind, (c and d) advection of anomalous moisture by the mean wind, and (e and f) advection of anomalous moisture by anomalous wind. The overbars denote climatology and primes denote deviation from climatology computed from the departure from the 1979-2022 climatology.

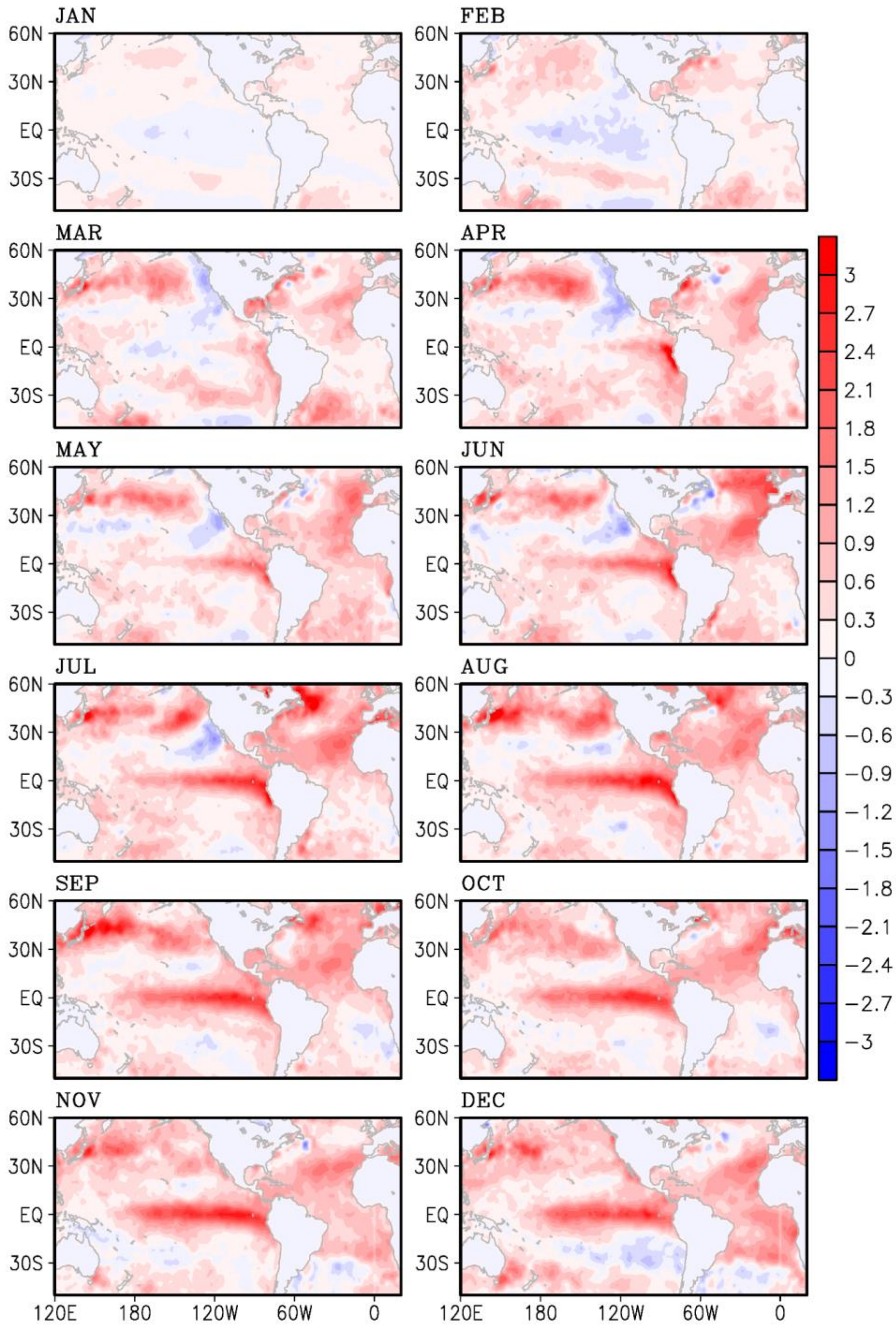

Supplementary Figure 8. Sea surface temperature anomalies (SSTA) prescribed to the atmospheric general circulation model experiment. These anomalies correspond to the year 2023. The amplitude of the anomalies was ramped up from climatology by prescribing one-third anomalies for January, two-third for February, and the full 2023 anomalies for the rest of the months (see Methods).

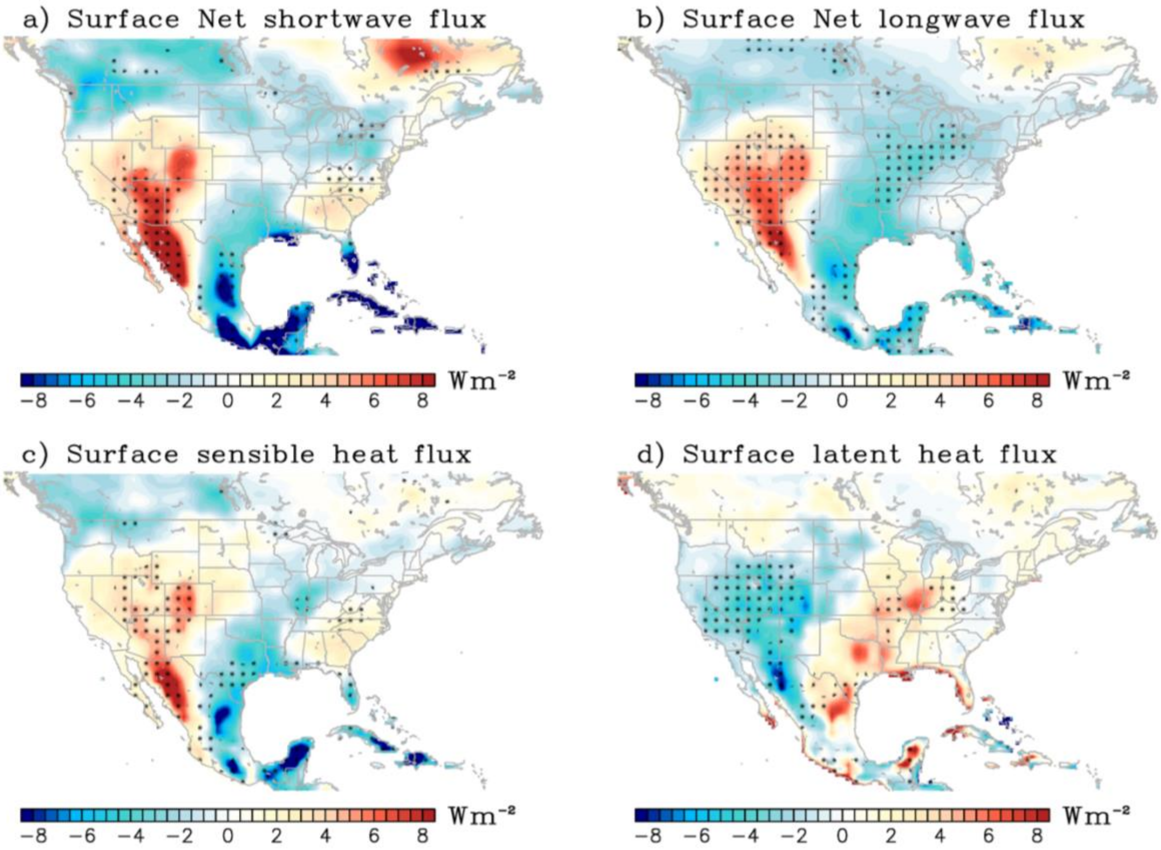

Supplementary Figure 9. Composite difference of simulated 2023 minus control atmospheric general circulation model (AGCM) experiments of surface heat fluxes during June-July-August for a) net shortwave radiation, b) net longwave radiation, c) sensible heat flux, and d) latent heat flux. The dot hatching indicates the 95% significance based on bootstrapping technique.

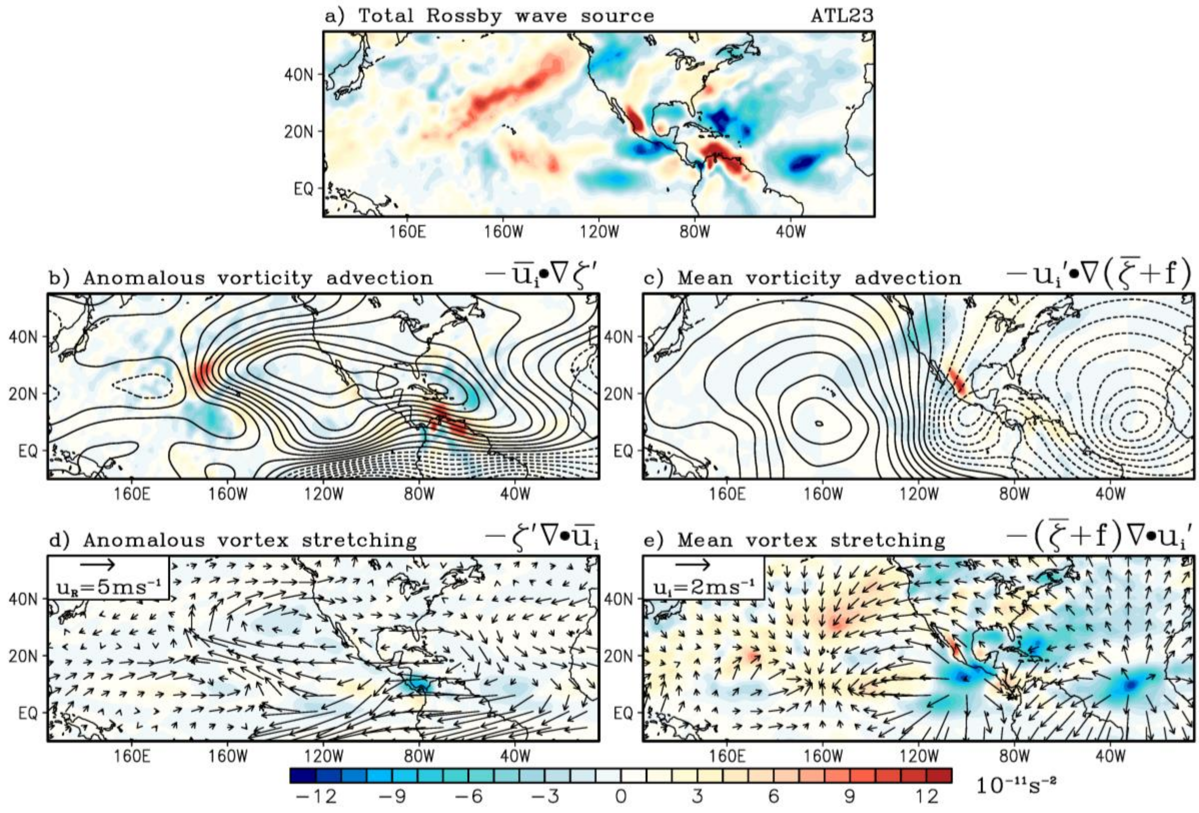

Supplementary Figure 10. Sea surface temperature (SST) sensitivity from the atmospheric model experiment with prescribed Atlantic 2023 SSTs (ATL23). Composite difference of simulated a) 200 hPa Rossby wave source (RWS), b) 200 hPa streamfunction (contour, $10^6\ s^{-1}$) and anomalous vorticity advection (color), c) 200hPa velocity potential (contour, $10^6\ s^{-1}$) and mean vorticity advection (color), d) 200 hPa rotational wind (vector, ms^{-1}) and anomalous vortex stretching (color), and e) 200hPa divergent wind component (vector, ms^{-1}) and mean vortex stretching. The units for the RWS terms are $10^{-11}\ s^{-2}$, see Methods for definition. Composites are from the AGCM experiment with prescribed 2023 Atlantic SSTs (ATL23). The composite differences are with respect to the control experiment (CTL) for June-July-August.

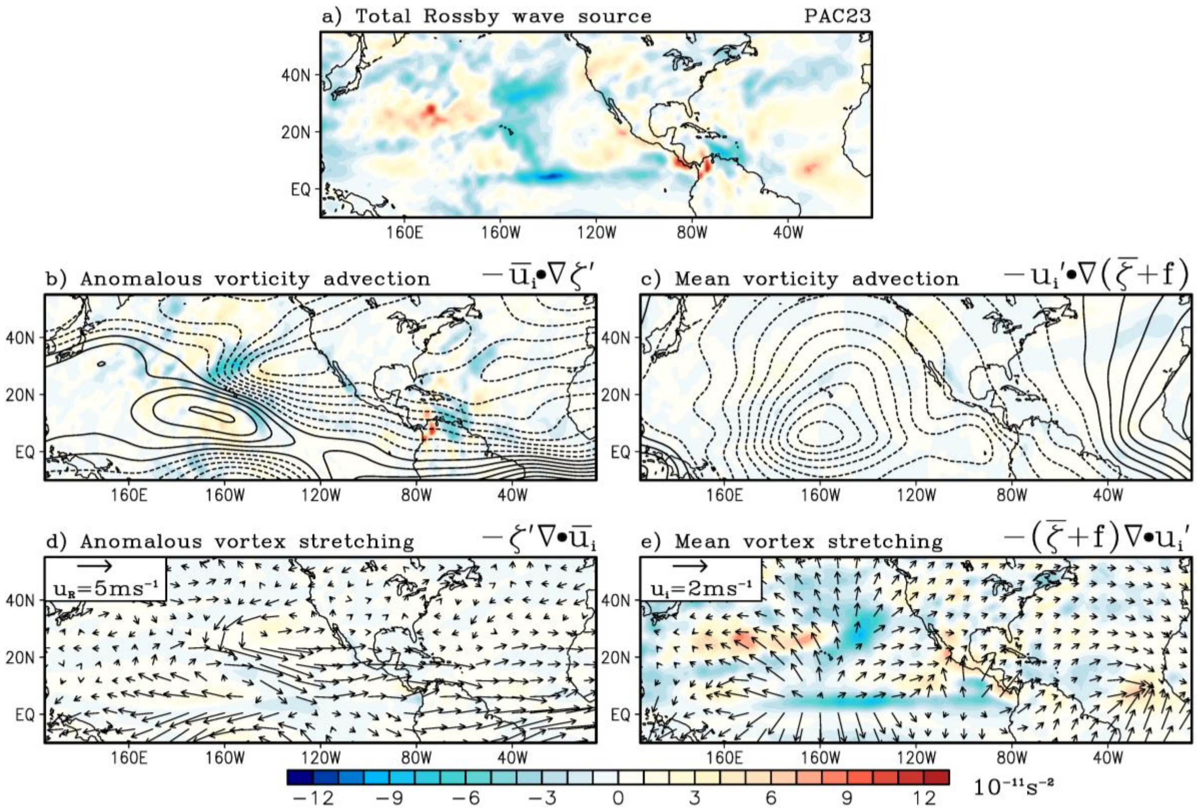

Supplementary Figure 11. Sea surface temperature (SST) sensitivity from the atmospheric model experiment with prescribed Pacific 2023 SSTs (PAC23). Composite difference of simulated a) 200 hPa Rossby wave source (RWS), b) 200 hPa streamfunction (contour, $10^6\ s^{-1}$) and anomalous vorticity advection (color), c) 200hPa velocity potential (contour, $10^6\ s^{-1}$) and mean vorticity advection (color), d) 200 hPa rotational wind (vector, ms^{-1}) and anomalous vortex stretching (color), and e) 200hPa divergent wind component (vector, ms^{-1}) and mean vortex stretching. The units for the RWS terms are $10^{-11}\ s^{-2}$, see Methods for definition. Composites are from the AGCM experiment with prescribed 2023 Pacific SSTs (PAC23). The composite differences are with respect to the control experiment (CTL) for June-July-August.

Supplementary Table 1. Changes in the mean and return period (days) of excess over a very high threshold temperature from the CTL (climatology), GBL23 (2023 SST sensitivity), ATL23 (Atlantic-only 2023 SST sensitivity), and PAC23 (Pacific-only 2023 SST sensitivity) AGCM experiments for JJA maximum and minimum temperatures for the grid-point closest to Phoenix, Arizona. The last row shows the Atlantic-Pacific synergy component (see Methods). The return periods are computed from a generalized Pareto distribution. Heat wave characteristics like, number of events, heat wave days, average (longest) duration in days, and average (strongest) amplitude temperature anomaly above the daily climatology temperature are shown.

	Maximum temperature		Minimum Temperature		Heat wave Characteristics			
	Mean (°C)	Return T=45°C	Mean (°C)	Return T=31°C	Number	Days	Duration (longest)	Amplitude (strongest)
CTL	39.1 ±0.30	45 years	25.8 ±0.31	30 years	77	307	3.9 (9) days	5.1 (7.7) °C
GBL23	40.2 ±0.31	14 years	26.8±0.34	11 years	184	853	4.6 (14) days	5.3 (8.3) °C
ATL23	39.7 ±0.31	19 years	26.6 ±0.33	10 years	135	581	4.3 (10) days	5.3 (8.8) °C
PAC23	39.2 ±0.31	31 years	25.8 ±0.33	29 years	101	391	3.9 (9) days	5.2 (8.2) °C
Syn*	39.5±0.31	32 years	26.0±0.33	43 years	102	495	4.2 (13) days	5.0 (6.7) °C
